# Influence of instrument design on post-operative pain in single-visit root canal treatment with Protaper Next and V taper 2H rotary systems in symptomatic irreversible pulpitis of multirooted teeth – A randomized clinical trial

**Published:** 2020-05-12

**Authors:** Koppala Sudhakar, Chinni Suneel Kumar, Anumula Lavanya, Sannapureddy Swapna

**Affiliations:** Department of Conservative Dentistry and Endodontics, Narayana Dental College and Hospital, Chintareddypalem, Nellore, Andhra Pradesh, India

**Keywords:** post-operative pain, protaper next, single-visit root canal treatment, symptomatic irreversible pulpitis, V taper 2H

## Abstract

**Aim:**

The objective of this randomized clinical trial was to clinically evaluate the incidence of postoperative pain after root canal treatment in symptomatic irreversible pulpitis of multirooted teeth using two different instruments design rotary file systems, namely, Protaper Next (PTN) and V taper 2H (VT2H).

**Materials and Methods:**

In this prospective randomized clinical trial, 60 patients with symptomatic irreversible pulpitis of multirooted teeth, indicated for root canal therapy, were randomly assigned to two groups according to instrument system used, namely, PTN and VT2H. Root canal treatments were performed in single visit. After treatment, the participants were asked to rate the intensity of post-operative pain on modified verbal descriptor scale after 8 h, 24 h, 48 h, and 72 h over the telephone by a second investigator. Patients were advised to call the second investigator by telephone if they felt very uncomfortable at any point of the follow-up time. At that time, they were asked to take ibuprofen 200 mg as the rescue drug.

**Results:**

No statistically significant difference was found among the two groups in relation to post-operative pain and intake of analgesic medication at 4 time points assessed (*P*>0.05, Kruskal–Wallis test).

**Conclusion:**

The PTN system and VT2H rotary system were found to be equal in the incidence of post-operative pain and pain intensity was found to decrease with time, postoperatively, in both the rotary systems.

**Relevance for patients:**

The instrument design has little effect on post-operative pain in symptomatic irreversible pulpitis patients after single-visit root canal therapy.

## 1. Introduction

Treating patients with symptomatic irreversible pulpitis in a single visit are a challenge as post-obturation pain is major concern. Post-operative pain is defined as any degree of pain that occurs after initiation of root canal therapy [1]. Varying degrees of post-operative pain have been observed in many clinical trials, ranging from 25% to 40% [2,3]. Greater incidence of post-operative pain has been observed in preoperatively symptomatic teeth than asymptomatic teeth [4]. A systematic review on pain prevalence and severity in root canal treated teeth observed that the incidence of post-operative pain was 40% in the first 24 h and decreased to 11% after 7 days [5].

Post-obturation pain is multi factorial in origin and can be influenced by gender, tooth type, insufficient instrumentation, irrigant extrusion, intracanal interappointment dressing extrusion, hyperocclusion, missed canals, presence of pre-operative pain, presence of periapical pathosis, apical debris extrusion, and apical patency during root canal preparation [6]. Another factor which may be a potential cause of post-obturation pain is the type of cleaning and shaping procedure which may result in extrusion of debris into periapical area [7,8].

The periapical extrusion of debris may induce an inflammatory reaction in the periapical area, leading to elevated concentrations of prostaglandins which usually manifests as pain. Hence, extrusion of infected debris to the periradicular tissues during cleaning and shaping is allegedly one of the principal causes of post-operative pain [9].

Many randomized clinical trials have noted a lesser incidence of post-operative pain with rotary systems when compared with reciprocation systems [10-12]. This observation has been validated by a recent meta-analysis by Hou *et al*. also [13]. The design of an instrument is an important factor determining apical extrusion of debris, which can contribute to post-operative pain [10].

The aim of the present randomized clinical trial was to clinically evaluate the post-obturation pain in single-visit root canal treatment with two different designs of instruments mainly Protaper Next (PTN) and V taper 2H (VT2H). The null hypothesis was that there is no difference in post-obturation pain after single-visit root canal treatment with PTN and VT2H rotary system in symptomatic irreversible pulpitis of multirooted teeth.

## 2. Materials and Methods

This parallel, double-blinded, randomized clinical trial was carried out after obtaining the approval of the Institutional Ethical Committee (reference no. D168401011) and registered under Clinical Trial Registry-India with register no. CTRI/2018/08/015339. The study subjects were recruited from the patients who registered in the department of conservative dentistry and endodontics.

### 2.1. Sample size

The minimum sample size required was determined to be 25 subjects per group to achieve 80% power to the study. The sample size was increased to 30 per group, to compensate for dropouts. A total of 60 patients out of which 18 male and 39 female patients of age between 18 and 45 years having multirooted teeth with symptomatic irreversible pulpitis, requiring endodontic therapy were included in the study.

### 2.2. Patient selection

Patients having multirooted teeth with symptomatic irreversible pulpitis were scheduled for endodontic treatment. Patients aged between 18 and 45 years, who showed prolonged response in the tooth even after the removal of the stimulus, who had the ability to understand the pain scales and informed consent for endodontic treatment were included in the study.

Patients with sinus tract, periapical abscess or facial cellulitis, known allergies, or any contraindication to opioid or non-opioid analgesics including aspirin or nonsteroidal anti-inflammatory drugs, patients with known allergy to local anesthesia, sodium hypochlorite and chlorhexidine, presence of systemic diseases such as cardiovascular disease, renal disease, and any bleeding disorders, and pregnant and nursing mothers were excluded from the study. Patients with more than 1 symptomatic tooth were excluded from the study.

### 2.3. Subjects allocation and randomization method

**Figure 1:**
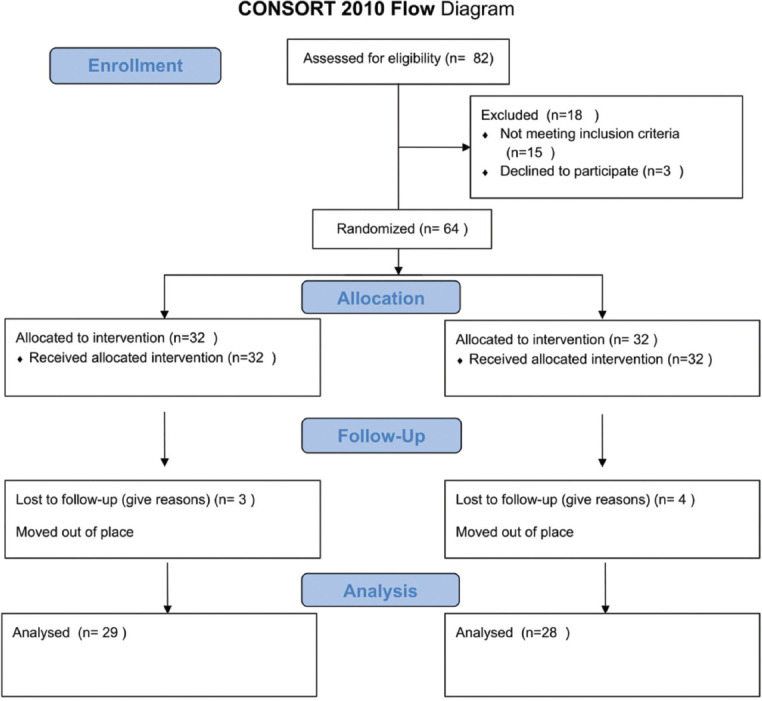
Consolidated standard of reporting trial CONSORT flow diagram.

Patients were assessed for eligibility criteria by an investigator not involved in the study. A pulpal and periapical diagnoses were given for each tooth on taking careful history, clinical and radiographic examination based on the American Association of Endodontists (AAE 2008) diagnostic guidelines. A total of 82 patients were assessed, among which 15 patients did not meet inclusion criteria, 3 patients refused to participate. Then, 64 eligible patients were divided into two groups by randomization with sequentially numbered opaque sealed envelopes (SNOSE) with 32 patients in PTN group and 32 patients in VT2H groups with allocation sequence and assigning to intervention which was done by blinded second investigator at the time of cleaning and shaping, concealed from operator to reduce bias. Out of 32 patients in PTN group, three patients were not interested in answering telephone calls so 29 patients were analyzed. Out of 32 patients in V2TH group, four patients were not interested in follow-ups and 28 patients were analyzed (Figure 1).

### 2.4. Treatment protocol

After taking case history, pre-operative pain levels were recorded using modified verbal descriptor scale (MVDS) system. Written informed consent was obtained from patients who participated in this study. All the teeth were treated by single operator. Endodontic therapy consisted of local anesthesia (Indoco Remedies Pvt. Ltd., Lignox 2%A, Mumbai, India) administration and rubber dam isolation followed by access cavity preparation. A size # 10 stainless steel hand K-file (Mani; Japan) was used to check the patency of canal. Root ZX II apex locator (J Morita Corp, Kyoto, Japan) was used to determine the working length which was further confirmed using periapical radiographs After working length determination, the canal was enlarged to size # 15 using stainless steel hand K-files (Mani; Japan). At this stage, randomization was carried out by second investigator based on SNOSE method and patients were assigned to two different rotary systems, namely, PTN (DENTSPLY Tulsa Dental Specialties, Tulsa, USA) and VT2H (SS White Dental, New Jersey) for cleaning and shaping. The canals were prepared with the files mounted on gear reduction handpiece powered by an electric motor (Endomate DT, NSK, Japan). The patient was blinded to the type of rotary system used for cleaning and shaping.

#### 2.4.1. PTN group

For the PTN group, Sx files (Protaper Universal system, Dentsply, Switzerland) were used for preflaring of coronal and middle thirds. X_1_ (17 0.4) and X_2_ (25 0.6) used for preparation of narrow and curved canals, using X_3_ (30 0.6) for wide canals up to working length. The files were used in continuous rotary motion at the speed of 300 rpm and torque 2 Ncm.

#### 2.4.2. VT2H group

For the VT2H group, glide path file, size 13 (0.3) and size 17 (0.4) were used for initial preparation, size 20 (0.6) and 25 (0.6) were used in narrow canals, size 30 (0.6) and size 35 (0.6) were used in wide canals up to the working length in continuous rotary motion at the speed of 300 rpm and torque 2 Ncm.

Irrigation was carried out using sodium hypochlorite and saline using side vented needles in both the groups. The final irrigation was completed with 2 ml of 2% chlorhexidine. After the final irrigation, canals were dried using absorbent paper points and obturated with 6% gutta-percha cone corresponding to apical preparation size and AH Plus (Dentsply Maillefer, Ballaigues, Switzerland) sealer after confirming master cone fit. Finally, the access cavity was sealed with a glass ionomer cement (GC Gold Label, Japan) liner followed by a composite restoration (Tetric N Ceram Ivoclar, Liechtenstein).

### 2.5. Assessment of post-operative pain and statistical analysis

All the participants received a card containing MVDS to assess pain levels after root canal treatment at 8 h, 12 h, 24 h, 48 h, and 72 h. According to this scale, the level of pain was documented in the range of 0-10 numerically and verbally as no pain (0), slight pain (1, 2), moderate pain (3-5), strong pain (6, 7), severe pain (8), and maximum pain (9, 10). All the participants were explained about the scale verbally before the start of the procedure.

Patients were contacted over telephone by the second investigator at 8, 24, 48, and 72 h time period and asked to describe the general feeling in the area of the treated tooth, pain intensity both numerical and verbal (MVDS), and intake of analgesics if any. The information so obtained was recorded at each follow-up period. None of the patients were prescribed with medication immediately after the treatment. They were asked to call the second investigator by telephone if they felt any discomfort in the treated area at any point of the follow-up time. If patient complained of pain, then they were prescribed with ibuprofen 200 mg as over-the-counter drug.

Statistical Package for the Social Sciences software was used for the statistical analysis. The Kruskal–Wallis non-parametric test was applied to compare the incidence of post-operative pain at the 4 time points assessed. The level of significance adopted was 5% (*P*<0.05).

## 3. Results

Baseline demographic and clinical features of study groups are summarized in Table 1.

**Table 1:** Base line demographic and clinical features

Sex and type of teeth	Protaper Next n, (%)	V taper 2H n, (%)	Total (%)
		
	*n*=29	*n* =28	*n*=57
Males	11 (37.9)	7 (25)	18 (32)
Females	18 (62.1)	21 (75)	39 (68)
Maxillary molars	14 (48.3)	15 (53.6)	29 (50.8)
Mandibular molars	14 (48.3)	12 (42.9)	26 (45.6)
**Maxillary premolars**	**01 (3.4)**	**01 (3.5)**	**02(3.5)**

Mean age of 57 patients enrolled in this study was 31 years and all the patients who underwent endodontic therapy answered the questionnaire satisfactorily at all the time points assessed (8 h, 24 h, 48 h, and 72 h). Out of 32 patients enrolled in PTN group, three patients were lost to follow up and 32 patients in VT2H group, four patients were lost to follow up. A total of 57 patients were analyzed with 29 in the PTN group and 28 in the VT2H group. There was no statistically significant difference (*P*>0.05) between the PTN and VT2H groups with regard to the incidence of post-operative pain at any of the 4 time points assessed (Table 2).

**Table 2:** Descriptive statistics for post-operative pain results in the groups instrumented with Protaper Next and V taper 2H.

Instrumentation system	*n*	Mean±standard deviation	Minimum	Maximum	25^th^ percentile	75^th^ percentile	*P* value
Pre-operative pain							
Protaper Next	29	5.86±1.382	4	8	5	6	0.503
V taper 2H	28	5.61±1.474	4	8	4	6	
Pain after 8 h							
Protaper Next	29	3.69±2.480	0	8	2	6	0.575
V taper 2H	28	4.07±2.624	0	8	2	6	
Pain after 24 h							
Protaper Next	29	2.00±1.813	0	6	0	3	0.940
V taper 2H	28	2.04±1.774	0	6	0	4	
Pain after 48 h							
Protaper Next	29	0.86±1.246	0	4	0	2	0.266
V taper 2H	28	1.29±1.584	0	6	0	2	
Pain after 72 h							
Protaper Next	29	0.55±0.985	0	4	0	1	0.647
V taper 2H	28	0.68±1.090	0	4	0	2	

**Figure 2:**
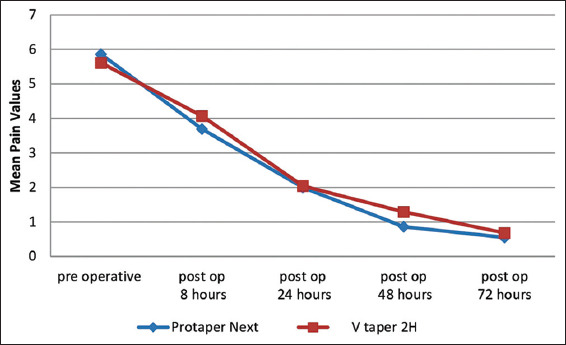
Decrease in pain score with increase in time period.

The highest mean post-operative pain scores were observed at 8 h follow-up time period in both the intervention groups with a significant decline thereafter (Figure 2).

In general, analgesics intake was confined to the first 24 h after treatment in the groups assessed. None of the patients in both the treatment groups needed analgesics at any time points assessed. None of the 57 participants reported severe pain or flare-ups during the period of study.

## 4. Discussion

Factors such as mechanical, chemical, microbial, immunological, gender, and psychological components may influence post-operative pain. Hence, in the present study, great care was taken to avoid all confounding factors and to minimize any unavoidable causes of post-operative discomfort. The distribution of males and females in both the groups was not statistically significant. Patients with two symptomatic teeth were excluded because pain from the one may influence the other.

The present study aimed to evaluate the incidence of post-operative pain in symptomatic multirooted teeth undergoing root canal treatment due to differences in instrument design. Studies have shown that different instrument designs may have different degree of apical debris extrusion. During cleaning and shaping, dentinal debris, necrotic pulp masses, irrigating solutions, and microorganisms from the root canal may gain access to apical periodontal tissues which leads to inflammation and post-operative pain that disturbs healing of periradicular tissues [9]. To minimize the role of medicaments and interappointment flare-ups, teeth that were indicated for single-visit root canal therapy were chosen for this study.

The age of the patients in our study ranged from 18 to 45 years in the PTN and VT2H groups. Out of 57 patients, 17 were male and 40 were female. In the PTN group, 10 were male and 17 were female, whereas in the VT2H group, 7 were male and 21 were female. There were no significant differences in age and gender distribution between the two groups, therefore, the effects of these variables were considered to be minimized.

To report the intensity of pain more precisely, Wang *et al*. chosen a MVDS, which is a combination of verbal descriptor scale of slight pain to maximum pain and normal rating scale of 0 for no pain to 10 for maximal pain. Hence, a MVDS has been employed in the present study [14]. In the present study, patients recruited in both the treatment groups were symptomatic with MVDS rating ranges from 3 to 8. Very few studies have assessed post-operative pain in symptomatic teeth with different instruments.

In general, pain levels that the patients experienced in our investigation were moderate to severe, that is, pain scores ranging from 4 to 8, with the highest MVDS score was observed in the PTN group and VT2H group at 8 h time interval. In both the groups, the highest mean MVDS scores were seen in 8 h subgroup after the root canal treatment. These results are consistent with findings of the previous clinical studies where the incidence of pain is maximum 8 h after the procedure [10,15]. In the present study, there was a no statistically significant difference in incidence of post-operative pain between the PTN group and VT2H group at 8 h, 24 h, 48 h, and 72 h after the root canal treatment. Even though in the both the groups post-operative pain levels were more at 8 h time period, there was no significant difference between these two groups at this time period.

It was observed that there was a gradual decrease in pain intensity in both groups with increasing time period. This decrease in tooth pain with increasing time is both logical and expected because this is a natural course of disease process after debridement. None of patients reported any other symptoms or complications such as post-operative swelling or paresthesia. All these facts highlight the level of care that was given to provide an atraumatic treatment protocol. In the present study, none of the patients in either of the treatment groups needed analgesics at any time period of the study.

Very few randomized clinical trials with pre-operative symptoms concluded that there is a reduction in post-operative pain over a period of time, irrespective of design of the rotary system used [15,16]. This study also confirms the same results.

The main parameter of a file that causes apical extrusion of debris is cross section and design of file [10]. Even though there is a difference in cross section and design of files used in the present study, there was no statistically significant difference in incidence of post-operative pain at 8 h, 24 h, 48 h, and 72 h after root canal treatment. A systematic review by Su have shown that short-term post-obturation pain, that is, immediate to 72 h time period is more with single-visit root canal therapy than multiple visit [17]. In the present study, the influence of instrument design between PTN and VT2H had little effect on post-obturation pain in both the treatment groups, with no significant difference in incidence of pain. Hence, the null hypothesis was accepted.

## 5. Conclusion

Incidence of post-operative pain was similar in symptomatic multirooted teeth prepared with PTN and VT2H rotary systems at all-time intervals. The intensity of pain was observed to be greater at 8 h with a gradual decrease in pain overtime in both the groups. None of the patients took analgesics in the observed post-operative period in both the groups.
